# Connexin 43 hemichannels and intracellular signaling in bone cells

**DOI:** 10.3389/fphys.2014.00131

**Published:** 2014-04-04

**Authors:** Lilian I. Plotkin

**Affiliations:** Department Anatomy and Cell Biology, Indiana University School of MedicineIndianapolis, IN, USA

**Keywords:** connexin43 hemichannels, osteoblast, osteocyte, apoptosis, bone

## Abstract

Cell function and survival are controlled by intracellular signals, and modulated by surrounding cells and the extracellular environment. Connexin channels participate in these processes by mediating cell-to-cell communication. In bone cells, gap junction channels were detected in the early 1970s, and are present among bone resorbing osteoclasts, bone forming osteoblasts, and osteocytes - mature osteoblasts embedded in the mineralized matrix. These channels are composed mainly by Cx43, although the expression of other connexins (45, 46, and 37) has also been reported. It is now believed that undocked Cx43 hemichannels (connexons) formed in unopposed cell membranes facing the extracellular environment participate in the interaction of bone cells with the extracellular environment, and in their communication with neighboring cells. Thus, we and others demonstrated the presence of active hemichannels in osteoblastic and osteocytic cells. These hemichannels open in response to pharmacological and mechanical stimulation. In particular, preservation of the viability of osteoblasts and osteocytes by the anti-osteoporotic drugs bisphosphonates depends on Cx43 expression *in vitro* and *in vivo*, and is mediated by undocked hemichannels. Cx43 hemichannels are also required for the release of prostaglandins and ATP by osteocytes, and for cell survival induced by mechanical stimulation *in vitro*. Moreover, they are required for the anti-apoptotic effect of parathyroid hormone in osteoblastic cells. This review summarizes the current knowledge on the presence and function of undocked connexons, and the role of hemichannel regulation for the maintenance of bone cell viability and, potentially, bone health.

## Introduction

**Figure 1:**
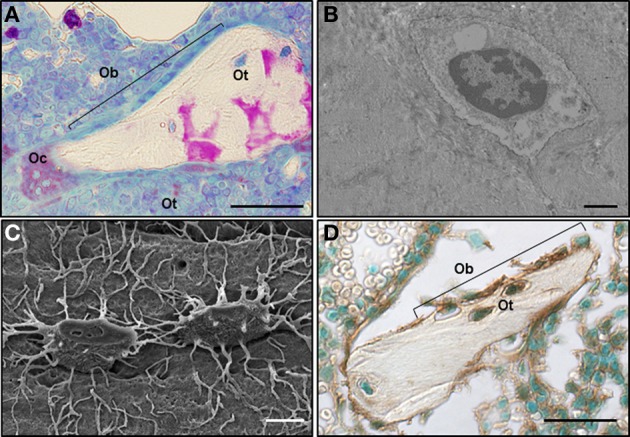
**Cell-to-cell interactions among osteoclasts, osteoblasts, and osteocytes in close proximity maintain bone homeostasis. (A)** An osteoclast (Oc), a team of osteoblasts (Ob) and bone-embedded osteocytes (Ot) are shown in a rat bone section, in which osteoclasts were stained for the osteoclast-specific enzyme tartrate resistant acid phosphatase (TRAPase) (red) and counterstained using Toluidine blue. Scale bar indicates 50 μm. Picture contributed by Keith W. Condon (Indiana University School of Medicine). **(B)** Transmission electron microscope image of an osteocyte embedded in the bone matrix. Image was obtained at the Electron Microscopy Center of the Department of Anatomy and Cell Biology (Indiana University School of Medicine). Scale bar indicates 5 μm **(C)** Scanning electron microscope image of an acid-etched bone showing two osteocytes highly communicated through their cytoplasmic projections (Bellido et al., 2014). Scale bar indicates 1 μm. **(D)** Cx43 immunostaining of a section of cancellous bone showing osteoblasts (Ob) on the bone surface and osteocytes (Ot) embedded in the bone matrix stained for Cx43 (brown) and counterstained with Methyl green (Plotkin et al., 2008). Scale bar indicates 50 μm.

The amount of bone and its strength is maintained throughout life by the concerted actions of osteoblasts, the bone forming cells, and osteoclasts, the bone removing cells (Figure 1A). The action of these two cell types is coordinated by signals derived from osteocytes, the bone resident cells that derived from osteoblasts and are embedded in the bone matrix (Figures 1A–D). Osteocytes are highly communicated among themselves and with cells of the bone surface through cytoplasmic projections (Figure 1C).

The existence of gap junctions between osteocytes and osteoblasts on the bone surface was first suggested by electron microscopy observations of calvaria bones from newborn mice (Weinger and Holtrop, 1974). Although the level of resolution of the images did not allow for these authors to conclusive demonstrate the presence of gaps separating the cell surfaces of osteoblasts and osteocytes, they concluded that the structures seen were consistent with gap junctions. This was later confirmed at the ultramicroscopy level by the groups of Doty (1981) and Marotti (Palumbo et al., 1990). Further studies showed that Cx43 is the most abundant connexin expressed *in vitro* and *in vivo* in all type of bone cells: osteoblasts, osteocytes and osteoclasts (Schirrmacher et al., 1992; Civitelli et al., 1993; Jones et al., 1993; Donahue et al., 1995). This is exemplified on a murine bone section stained for Cx43 (Figure 1D).

The small molecules that are transferred through connexin channels, and might act as second messengers in bone cells have not been completely identified (see Stains et al., 2014 for a recent revision). Second messengers such as ATP and Ca^2+^ can move from one cell to another through gap junctions, or can be released to the extracellular media through hemichannels in osteoblastic cells (Jorgensen et al., 1997; Genetos et al., 2007). In addition, cAMP production induced by parathyroid hormone requires Cx43 expression in osteoblastic cells (Vander Molen et al., 1996; Bivi et al., 2011), and Cx43-mediated amplification of FGF2 effect on the osteoblast gene RUNX2 depends on the production of water-soluble inositol polyphosphates (Niger et al., 2013). Taken together, these pieces of evidence suggest that Cx43 not only can control the movement of second messengers, but also their synthesis.

The expression of Cx45, Cx46 and, more recently, Cx37 has also been demonstrated in bone cells (Kruger et al., 2000; Stains and Civitelli, 2005; Paic et al., 2009; Chaible et al., 2011; Pacheco-Costa et al., 2014). In particular, Cx37 is required for osteoclast differentiation and mice lacking Cx37 exhibit high bone mass due to defective bone resorption (Pacheco-Costa et al., 2014).

In addition to be part of gap junctions, connexin channels can be found in unopposed cell membranes forming undocked connexons or hemichannels. Although it was long known that bone cells express connexins, the presence of hemichannels in osteoblastic cells was not reported until 2001 (Romanello and D'Andrea, 2001). In the current review, the demonstration of the presence and function of connexin hemichannels in osteoblasts and osteocytes is discussed.

## Cx43 and bone development: a role for hemichannels?

The importance of Cx43 expression in osteoblasts and osteocytes for bone development, as well as for osteoblast and osteocyte differentiation, survival and function has been clearly established (for recent reviews see Civitelli, 2008; Loiselle et al., 2013; Plotkin and Bellido, 2013). Thus, global deletion of Cx43 results in delayed ossification and impaired osteoblast differentiation in the embryos (Lecanda et al., 2000). In addition, studies with tissue specific deletion of Cx43 have demonstrated that the adult skeleton is also affected by the absence of the connexin (Chung et al., 2006; Watkins et al., 2011; Zhang et al., 2011; Bivi et al., 2012a,b). Cx43 is also important for osteoclast differentiation, as demonstrated in mice in which the connexin was deleted from osteoclast precursors (Sternlieb et al., 2012). Because these studies were performed by deleting the whole Cx43 molecule, it is not possible to determine whether absence of cell-to-cell gap junction communication or the function of Cx43 in undocked hemichannels present in cell membranes (or even channel-independent functions of the connexin), or a combination of these functions, are responsible for the observed phenotypes. Nevertheless, recent developments discussed below support the presence and functionality of Cx43 hemichannels in bone cells *in vivo*.

Mutations of the Cx43 gene are associated with occulodentodigital dysplasia (ODDD), a condition with skeletal malformation that include craniofacial abnormalities and broad long bones (Paznekas et al., 2002). Interestingly, some of the Cx43 mutations leading to ODDD result not only in decreased gap junction communication, but also in enhanced hemichannel activity (Dobrowolski et al., 2007), suggesting that part of the phenotype of the patients might be due to exacerbated hemichannel function. Consistent with this, a study reported in the form of an abstract showed that osteocytic expression of the Cx43 mutant R76W, which does not form gap junctions but has the ability to form hemichannels, leads to decreased bone mineral density (Jiang et al., 2010). The phenotype of these mouse models might be due to the lack of intercellular gap junction communication or, alternatively, to excessive hemichannel activity in bone cells, resulting in skeletal defects.

Interestingly, stimuli that increase bone mass have been shown to increase Cx43 expression, its localization in the cell membrane, and the activation of gap junction and hemichannel activity. Thus, estrogen, increases the expression of Cx43 and gap junction communication in the osteocytic cell line MLO-Y4 (Ren et al., 2013); and the effect of sex steroid removal on the cortical bone is partially prevented in mice lacking Cx43 in osteoblastic cells (Watkins et al., 2012). However, we have shown that the anti-apoptotic effect of sex steroids on osteocytic cells does not require Cx43 function (Plotkin et al., 2002). On the other hand, as it will be discussed below, Cx43, and in particular, hemichannels, mediate at least in part the effect of the anti-osteoporotic drugs bisphosphonates (Plotkin et al., 2002, 2008), mechanical stimulation (Cherian et al., 2005; Zhang et al., 2011; Grimston et al., 2012; Bivi et al., 2013), and parathyroid hormone (Bivi et al., 2011).

## The bone protecting drugs bisphosphonates preserve osteoblast and osteocyte viability by opening Cx43 hemichannels

**Figure 2:**
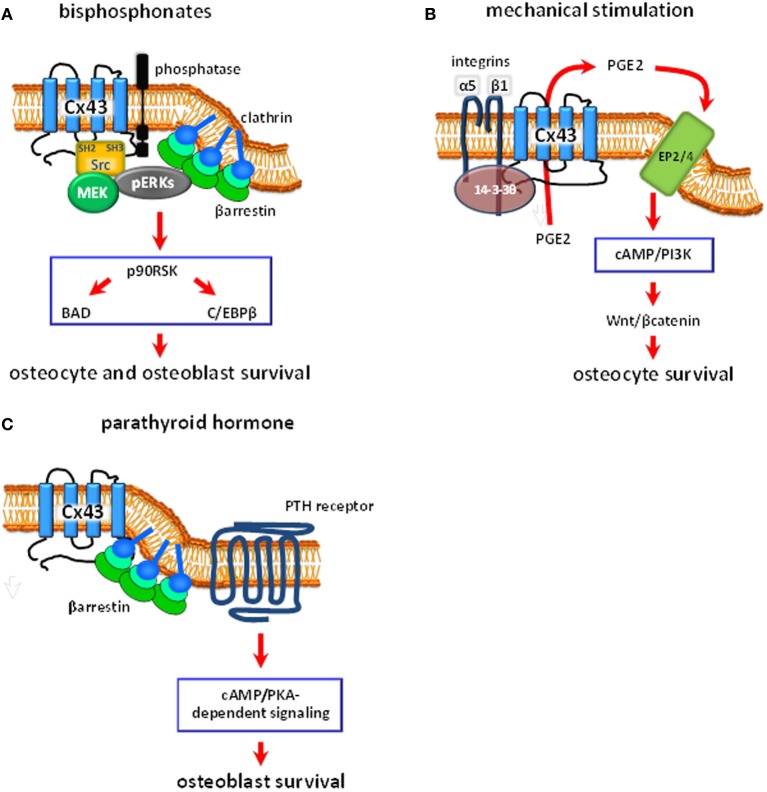
**Schematic representation of the proposed intracellular signaling pathways regulated by Cx43 hemichannels in bone cells. (A)** Bisphosphonates bind to phosphatases present in the cell membrane. This induces Cx43 hemichannel opening, followed by activation of the kinases Src and MEK, and ERKs. ERKs activated downstream of Cx43 hemichannel opening are retained in the cytoplasm by a complex formed by βarrestin and clathrins. This leads to the phosphorylation of the cytoplasmic targets p90^RSK^, BAD and C/EBPβ, which results in osteoblast and osteocyte survival. **(B)** Mechanical stimulation induces α5β1 integrin engagement and the association of the integrins with Cx43, by a mechanism that requires the protein 14-3-3θ. This results in hemichannel opening and the release of PGE2. PGE2, in turn, activates EP2/4 prostaglandin receptor by an autocrine/paracrine mechanism, leading to activation of the cAMP/PI3K signaling pathways, accumulation of βcatenin with the consequent activation of Wnt signaling, and inhibition of osteocyte apoptosis. **(C)** Parathyroid hormone (PTH), through binding to the PTH receptor, induces activation of the cAMP/PKA signaling pathway. Cx43, by sequestering β-arrestin away from the PTH receptor, facilitate cAMP/PKA-mediated downstream signaling and osteoblast survival.

Bisphosphonates, a family of drugs that include alendronate, have been used over the past 40 years to treat conditions with low bone mass such as osteoporosis, and to prevent bone fractures (Russell, 2011). Bisphosphonates block osteoclastic bone resorption, therefore preserving the amount of bone. However, stopping bone resorption cannot completely explain the ability of these drugs to prevent bone fractures. We therefore proposed that, in addition to inhibiting bone resorption, bisphosphonates have a positive effect on osteoblasts and osteocytes that can contribute to the anti-fracture properties of the drugs. Preservation of osteoblast life span should lead to prolonged matrix synthesizing activity, whereas maintenance of osteocyte viability should preserve their mechanosensory function, therefore improving bone strength. We found that bisphosphonates protect osteoblasts and osteocytes from apoptosis induced by several agents (Figure 2A) *in vitro* using osteoblastic cell isolated from neonatal calvaria bone and osteocytic MLO-Y4 cells, and by glucocorticoid excess *in vivo* using a mouse model of glucocorticoid-induced bone disease (Plotkin et al., 1999). Although osteoblasts and osteocytes have distinct functions, they respond in similar fashion to bisphosphonates. Therefore, the studies described in this section were performed with both cell types.

The survival effect of bisphosphonates is mediated by the activation of the extracellular signal regulated kinases ERKs in cell cultures and *in vivo* (Figure 2A) (Plotkin et al., 1999, 2011). Thus, phosphorylation of ERKs is increased upon treatment of cells or mice with the bisphosphonate alendronate. Importantly, ERK activation is required for the protective effect of bisphosphonates since their effect was abolished by ERK pharmacological inhibitors and by transfection of a dominant negative form of MEK, the kinase that activates ERKs (Plotkin et al., 1999, 2002).

ERKs can be activated by an array of extracellular stimuli, including binding of growth factors and cytokines to their receptors. We then explored the possibility that bisphosphonates activate ERKs by acting on membrane channels. Because connexin channels are abundant in osteoblasts and osteocytes, and bisphosphonates are molecules of low molecular size (approximately 400 daltons), we examined whether inhibition of connexin channels affected the protective response to the bisphosphonate alendronate. We found that inhibiting connexin channels with 18 α-glycyrrhetinic acid (AGA) abolished the anti-apoptotic effect of alendronate, whereas the inactive analog of AGA, glycyrrizic acid (GA), did not modify this response (Plotkin et al., 2002). Similar to anti-apoptosis, AGA, but not GA, prevented ERK activation induced by alendronate. These results suggested that the connexin channels are required for the ERK-mediated anti-apoptotic effect of bisphosphonates. However, prevention of apoptosis by alendronate does not require cell-to-cell contact, since the bisphosphonate inhibited apoptosis in cells plated at low density or maintained in suspension, suggesting that gap junctions do not mediate the effect of bisphosphonates and that hemichannels are involved. Moreover, treatment with alendronate did not affect gap junction communication. Instead, alendronate induced opening of hemichannels in MLO-Y4 cells measured as uptake of the fluorescent small dye Lucifer Yellow. Because Cx43 is the main connexin protein expressed in osteocytic MLO-Y4 cells, we next determined whether responsiveness to alendronate depends on its expression using several cell models (Plotkin et al., 2002). We found that the drug was able to protect from apoptosis embryonic fibroblasts derived from wild type mice, but it was unable to do so in cells derived from Cx43 deficient mice, and that transfecting Cx43 to deficient cells rescued responsiveness to alendronate. Similarly, ROS17/2.8 osteoblastic cells that express Cx43 were protected from apoptosis by alendronate, but UMR 106 cells which do not express Cx43 were not. And Cx43 transfection to UMR106 cells rescued responsiveness to alendronate. Moreover, authentic osteoblasts derived from Cx43 deficient mice did not respond to alendronate, whereas apoptosis was prevented by this agent in osteoblasts derived from wild type mice. Cx43, but not other connexins tested as representative of the three connexin subfamilies (26, 31, 32, 37, 40, and 45), was able to confer responsiveness to alendronate in HeLa cells, which do not express connexins (Plotkin et al., 2002). These results established the requirement of Cx43 for the anti-apoptotic effect of bisphosphonates *in vitro*.

The cytoplasmic C-terminal domains of connexins differ considerably, suggesting that the specific requirement of Cx43 for the effect of alendronate could be due to this region. Indeed, a Cx43 truncated mutant that lacks the cytoplasmic tail was unable to confer responsiveness to alendronate, response that was recovered by co-transfecting the truncated mutant and the cytoplasmic tail (Plotkin et al., 2002). However, the C-terminus tail alone was not able to mediate anti-apoptosis. These results suggest that the C-terminus of Cx43 contains domains required for anti-apoptosis by bisphosphonates and that the pore-forming region also contributes to the effect.

Further studies showed that bisphosphonates induce the transient opening of undocked Cx43 hemichannels resulting in activation of Src kinase, which is associated with Cx43, leading to ERK activation and cell survival (Figure 2A) (Plotkin et al., 2002). Since receptors for bisphosphonates have not been described, the requirement of Cx43 for bisphosphonate actions raised the possibility that Cx43 would be such receptor. However, we found that bisphosphonates not only bind to cells that express Cx43 but also to Cx43-deficient cells (Lezcano et al., 2012). Moreover, binding of labeled bisphosphonate to osteoblastic cells is displaced by unlabeled bisphosphonates, as expected, but also by protein phosphatase substrates. Furthermore, these substrates inhibit the anti-apoptotic effect of bisphosphonates (Morelli et al., 2011) Changes in the phosphorylation status of Cx43 C-terminus tail are associated with channel activity (reviewed in Herve and Sarrouilhe, 2006), suggesting that bisphosphonates might induce opening of Cx43 hemichannels thought their interaction with membrane bound phosphatases. However, the identity of the protein phosphatase that binds to Cx43, and whether it can dephosphorylate Cx43 and induce hemichannel opening remain to be determined. Taken together, these pieces of evidence indicate that although Cx43 is required for survival signaling, it is dispensable for bisphosphonate binding; and raises the possibility that bisphosphonates bind to a protein phosphatase that interact with Cx43 in the cell membrane.

An interesting feature of ERKs activated by bisphosphonates and the Cx43/Src pathway is that instead of undergoing nuclear translocation like ERKs activated by most stimuli, they are retained in the cytoplasm where ERKs modify cytoplasmic substrates (Plotkin et al., 2005a). We found that this phenomenon is due to the ability of Cx43 of interacting with β-arrestins (Plotkin et al., 2006b). Consistent with a role of β-arrestin in the retention of bisphosphonate-activated ERKs in the cytoplasm, ERKs stay in the cytoplasm in cells expressing endogenous β-arrestin or transfected with wild type β-arrestin. However, in the presence of a dominant negative from of β-arrestin, ERKs activated by alendronate translocate to the nucleus. Because cytoplasmic localization of ERKs is required for survival induced by bisphosphonates (Plotkin et al., 2005a), the dominant negative β-arrestin reversed anti-apoptosis induced by alendronate. Thus, Cx43 regulates the ERK signaling pathway due to its ability of function as a scaffold that foster interaction between β-arrestin and Src/ERKs.

Further support for the role of Cx43 on the survival effect of bisphosphonates was obtained *in vivo*, by the demonstration that administration of alendronate does not prevents glucocorticoid-induced osteoblast and osteocyte apoptosis in mice lacking Cx43 in these cells (Plotkin et al., 2008). However, the bisphosphonate was still able to prevent bone loss induced by glucocorticoids due to its potent inhibition of bone resorption. Similarly, bisphosphonates prevented bone loss induced by sex steroid removal in mice lacking Cx43 in osteoblasts and osteocytes, by reducing bone resorption (Watkins et al., 2012). On the other hand, IG9402, a bisphosphonate that does not affect osteoclast function by prevents osteoblast and osteocyte apoptosis, prevents bone loss induced by glucocorticoids in mice (Plotkin et al., 2006a, 2011). This suggest that in the absence of anti-resorptive effects, the protective effect of preventing osteocyte and osteocyte apoptosis through opening Cx43 hemichannels can be revealed. Thus, preservation of osteoblast and osteocyte apoptosis mediated by hemichannel opening might contribute to the anti-fracture efficacy of bisphosphonates.

## Mechanical stimulation opens Cx43 hemichannels *in vitro*, a mechanism that mediates cell survival induced by mechanical forces

Adequate mechanical stimulation is required for maintaining bone mass and strength throughout life. Indeed, reduced or absent loading leads to decrease bone mass and elevated risk of fractures in the elderly, in immobilized patients, and in astronauts (Bikle et al., 1997; Marcus, 2002; Dolbow et al., 2011). Osteocytes are ideally positioned for detecting and responding to changes in mechanical strains imposed on bone. Since their description at the microscopy level, connexin channels were thought to be the means by which the cells buried in the bone communicate with the cells on the bone surface and transmit the need for bone formation or removal.

Consistent with a role of connexin channels on mechanotransduction in bone, Cx43 expression and intercellular communication is increased by loading *in vitro* and *in vivo* (Ziambaras et al., 1998; Cheng et al., 2001b; Robinson et al., 2006; Tu et al., 2012). Early work by Donahue's group show that mechanical stimulation of osteoblastic cells induced by oscillatory fluid flow leads to release of prostaglandin (PG) E2 by a mechanism that requires intact Cx43 channels (Figure 2B) (Saunders et al., 2001). Thus, the release of PGE2 was inhibited in cells expressing a dominant negative form of Cx43 with reduced channel permeability. Moreover, PGE2 release induced by mechanical stimulation further increased gap junction communication (Cheng et al., 2001a) by a mechanism mediated by the prostaglandin EP2 receptor (Cherian et al., 2003) suggesting the existence of a positive feedback loop. This effect of mechanical loading was originally ascribed to a role of Cx43 in intercellular gap junction communication. However, based on previous studies showing that mechanical stimulation increases hemichannel opening in osteoblastic cells *in vitro* (Romanello et al., 2003), work from Jiang's group demonstrated that PGE2 released induced by mechanical stimulation requires opening of Cx43 hemichannels in osteocytic cells (Figure 2B) (Cherian et al., 2005). Interestingly, hemichannels present in the osteocytic cell body, and not in the dendritic cytoplasmic projections, are opened by mechanical stimulation (Burra et al., 2010). The release of PGE2 induced by fluid flow in osteocytic MLO-Y4 cells was prevented by using pharmacologic inhibitors of connexin channels and by an anti-sense oligonucleotide for Cx43 (Cherian et al., 2005). Moreover, prostaglandin release was enhanced when the cells were seeded at low density, which reduces intercellular gap junction communication. Intracellular levels of PGE2 production were not affected by inhibition of connexin channels, suggesting that PGE2 release but not its synthesis requires Cx43 hemichannel activity. Other authors have postulated, however, that P2X7 receptors (Li et al., 2005) and/or pannexin1 (Thi et al., 2012) are involved in the release of PGE2 induced by mechanical stimulation in bone cells. This suggests that the combined actions of these channels are required for prostaglandin release induced by mechanical stimulation in osteoblastic and osteocytic cells.

Further studies by Jiang's group demonstrated that engagement of α5β1 integrin in required for opening of Cx43 hemichannels in osteocytic cells (Figure 2B) (Batra et al., 2012). Thus, Cx43 interacts with α5β1 integrin, an interaction enhanced by mechanical stimulation (Batra et al., 2012, 2014). Cx43/α5β1 association is required for Cx43 hemichannel opening induced by loading and is mediated by activation of phosphatidylinositol-3 kinase (PI3K) and AKT. Consistent with a role of the integrins on mechanotransduction, we have shown that mechanical stimulation leads to the engagement of integrins α5 and β1, which in turn activate FAK/Src and the ERK pathway promoting osteocyte survival (Plotkin et al., 2005b).

More recently, Jiang's group showed that the scaffolding molecule 14-3-3θ is required for the interaction between integrin α5 and Cx43 and for plasma membrane delivery and function of Cx43 hemichannels (Figure 2B) (Batra et al., 2013). In particular, silencing of 14-3-3θ prevents the accumulation of Cx43 on the cell membrane and opening of hemichannels induced by fluid flow. Taken together, these studies support a role of Cx43 hemichannels on PGE2 release.

PGE2 release by osteocytes through Cx43 hemichannels subjected to mechanical forces is required for maintaining cell viability (Figure 2B). Indeed, prostaglandins prevent osteoblastic apoptosis via activation of EP4 receptors (Machwate et al., 2001); and mechanical stimulation prevents osteocyte apoptosis *in vitro* (Plotkin et al., 2005b; Aguirre et al., 2007; Kitase et al., 2010). Moreover, inhibition of glucocorticoid-induced apoptosis by mechanical stimulation is abolished by inhibiting prostaglandin synthesis using indomethacin (Kitase et al., 2010). The pro-survival effect of PGE2 release by osteocytes subjected to mechanical stimulation in mediated by activation of the EP2 and EP4 receptors, via cAMP/PKA signaling pathway. In addition, the study by Kitase and colleagues showed that activation of Wnt/βcatenin signaling downstream of PI3K/Akt contributes to PGE2/EP2/4-induced osteocyte survival (Figure 2B).

The participation of the Cx43/PGE2 survival pathway in skeletal homeostasis *in vivo* is not known. However, this potential role of Cx43 is supported by our work (Plotkin et al., 2008; Bivi et al., 2012a), later confirmed by others (Lloyd et al., 2013) showing that deletion of Cx43 from osteoblastic cells results in increased osteocyte apoptosis. Interestingly, recent work from Jiang's group (Xu et al., 2013) showed that transgenic mice expressing in osteocytes a dominant negative Cx43 mutant with impaired permeability (Cx43Δ130-136), which lacks both hemichannel and gap junction functions (Krutovskikh et al., 1998), also exhibit increased osteocyte apoptosis (Jean X. Jiang, personal communication). On the other hand, mice with osteocytic expression of the R76W Cx43 mutant, in which the ability to form gap junction channels is impaired, but that maintains hemichannel activity (Xu et al., 2013) did not exhibit increased osteocyte apoptosis compared to wild type controls (Jean X. Jiang, personal communication). This evidence supports the role of Cx43 hemichannels for maintain osteocyte survival *in vivo*; and suggests that in the absence of Cx43 hemichannels in osteocytes, the mechanical loading that occurs during normal ambulatory conditions cannot protect the cells from undergoing apoptosis. Indeed, one of the earliest effects of skeletal unloading is the accumulation of apoptotic osteocytes (Aguirre et al., 2006).

However, Cx43 is not required for the increased bone mass induced by mechanical stimulation in murine models. On the contrary, removal of Cx43 from osteoblasts and/or osteocytes results in increased anabolic response to mechanical stimulation of osteoblast on the periosteal bone surface (Zhang et al., 2011; Grimston et al., 2012; Bivi et al., 2013). The cause of this exacerbated response is not known. We have found that Cx43-deficient osteocytes exhibit elevated Wnt/βcatenin signaling (Bivi et al., 2013), a known mediator of mechanical signals in osteocytes (Robinson et al., 2006). This higher basal activation of Wnt signaling could explain the exacerbated response to loading in Cx43-deficient mice. Nevertheless, mounting evidence indicates that Cx43 hemichannel opening and the release of PGE2 mediate the survival effect of mechanical stimulation on osteocytes, suggesting that the increase in bone formation induce by mechanical stimulation depends on signaling pathway different from those involved in loading-induced osteocyte survival.

## Cx43 expression and channel permeability but not gap junction channels are required for cAMP-mediated anti-apoptotic effect of parathyroid hormone on osteoblasts

Intermittent administration of parathyroid hormone (PTH) is the only FDA-approved treatment to increase bone mass. Cx43 expression appears to be required to obtain a full anabolic response to intermittent PTH administration in mice (Castro et al., 2003; Chung et al., 2006). Thus, PTH does not increase bone mass, bone formation and osteoblast number when administered to heterozygous Cx43 deficient mice (Cx43^+/−^) (Castro et al., 2003). Moreover, PTH-induced mineral appositional rate, a measure of the work of osteoblast teams, is reduced in mice lacking Cx43 in osteoblastic cells (in Cx43^fl/−^; Col1a1-2.3kb-Cre mice) (Chung et al., 2006). Studies in mice have shown that the anabolic effect of intermittent PTH administration in cancellous bone is due, at least in part, to inhibition of osteoblast apoptosis (Figure 2C) (Jilka et al., 1999). Similarly, PTH related protein (PTHrP), the other ligand of the PTH receptor, as well as constitutive activation of this receptor in transgenic mice, also increases osteoblast number and decreases the prevalence of osteoblast apoptosis (Calvi et al., 2001; Martin, 2005; Miao et al., 2005; O'Brien et al., 2008). Taken together, these pieces of evidence suggest that intermittent PTH administration does not result in a full anabolic response in mice lacking Cx43 due to the inability of the hormone to prevent apoptosis of osteoblastic cells in the absence of Cx43.

*In vitro* mechanistic studies showed that, indeed, PTH does not prevent apoptosis in osteoblastic cells lacking Cx43, whereas overexpression of wild type Cx43 rescues the survival effect of the hormone (Figure 2C) (Bivi et al., 2011). A similar result is obtained by transfecting a Cx43 mutant in which cysteine residues of the extracellular domain have been mutated, rendering it unable to dock with other connexin molecules to form gap junction channels (Bao et al., 2004). On the other hand, a Cx43 mutant with impaired permeability was not able to confer responsiveness to PTH, suggesting that active Cx43 hemichannels are required for the survival effect of the hormone.

In addition, the interaction of Cx43 with β-arrestin modulates the response of osteoblasts to PTH (Figure 2C) (Bivi et al., 2011). Thus, PTH-induced anti-apoptosis is rescued by transfecting a dominant negative form of β-arrestin to cells lacking Cx43; and overexpression of β-arrestin induces the same inhibition on PTH-induced osteoblast survival as removing Cx43. Moreover, transfection of Cx43 decreases the association between the PTH receptor and β-arrestin, suggesting that Cx43 binds to β-arrestin, thus competing with the PTH receptor. Interestingly, Cx43 mutants lacking the C-terminus domain or lacking the phosphorylation site in serine 368 decrease the interaction between PTHR and β-arrestin, suggesting that Cx43 binds β-arrestin at the phosphorylated serine 368.

We found that PTH does not increase the expression of cAMP-target genes in osteoblastic cells lacking Cx43 (Bivi et al., 2011). Consistent with this, it was previously shown that the response to PTH on cAMP production is blunted in osteoblastic cells in which Cx43 expression has been reduced using anti-sense cDNA (Vander Molen et al., 1996). These findings, together with evidence that β-arrestin reduces cAMP responses of the PTH receptor (Premont and Gainetdinov, 2007), support the hypothesis that Cx43 interacts with β-arrestin decreasing β-arrestin binding to the PTH receptor, then facilitating cAMP dependent signaling induced by PTH. Thus, Cx43 hemichannel activity and the ability of the connexin to interact with intracellular signaling molecules, through specific phosphorylation sites in its cytoplasmic tail, regulates survival signaling induced by PTH in osteoblastic cells.

## Conclusions

Since the first description of gap junction channels at the structural level in bone cells, substantial advances on our understanding of the role of connexins on bone cell physiology have been made (Civitelli, 2008; Loiselle et al., 2013; Plotkin and Bellido, 2013). The requirement of connexins, and in particular Cx43, for bone cell differentiation and function, as well as for the response of the cells to bone-acting stimuli has been clearly established *in vitro* and *in vivo*. Moreover, the role of the Cx43 hemichannels *in vivo* has begun to be unveiled. Indeed, while studies with genetically modified mice appear to agree that sustained opening of Cx43 hemichannels is deleterious for bone (Dobrowolski et al., 2007; Jiang et al., 2010), transient opening of hemichannels is beneficial for osteoblast and osteocyte survival (Plotkin et al., 2002, 2008, 2011; Kitase et al., 2010; Bivi et al., 2011). Studies using genetically modified mice currently underway will allow to conclusively demonstrate the role of Cx43 hemichannels on bone development and cell function.

### Conflict of interest statement

The author declares that the research was conducted in the absence of any commercial or financial relationships that could be construed as a potential conflict of interest.
